# Landscape and Local Drivers Affecting Flying Insects along Fennel Crops (*Foeniculum vulgare*, Apiaceae) and Implications for Its Yield

**DOI:** 10.3390/insects12050404

**Published:** 2021-04-30

**Authors:** Lucie Schurr, Benoît Geslin, Laurence Affre, Sophie Gachet, Marion Delobeau, Magdalena Brugger, Sarah Bourdon, Véronique Masotti

**Affiliations:** 1 Aix Marseille Univ, Univ Avignon, CNRS, IRD, IMBE, Campus Étoile, Faculté des Sciences St-Jérôme, Case 421 Av Escadrille Normandie Niémen, CEDEX 20, 13397 Marseille, France; benoit.geslin@imbe.fr (B.G.); laurence.affre@imbe.fr (L.A.); sophie.gachet@imbe.fr (S.G.); delobeau.marion@gmail.com (M.D.); magdalena.brugger@outlook.fr (M.B.); sarah.bourdon@etu.univ-amu.fr (S.B.); veronique.masotti@imbe.fr (V.M.); 2 Tours Univ, Polytech Tours, 35, Allée Ferdinand de Lesseps, 37200 Tours, France; 3 Paris-Saclay Univ, Bât. 300, 15 rue Georges Clémenceau, CEDEX, 91405 Orsay, France

**Keywords:** fennel, semi-natural habitat, interspersion and juxtaposition index (IJI), insecticides–insect abundance and richness–essential oil yield

## Abstract

**Simple Summary:**

In a globally strained context where food production constantly challenges biodiversity, the importance of insect activity to crop pollination is at stake, as insects are essential to more than 75% of global crops. Despite this awareness, there is still a gap of knowledge about the importance of pollinators for aromatic crops. Fennel is an aromatic plant cultivated in the South of France for its essential oil, which is of great economic interest. Here, we explored the effect of the abundance and richness of insects caught at the edge of fennel crops on the yield of essential oil. We found that high insect richness improves fennel essential oil yield. In this context, it appeared important to know what structured the insect communities we trapped. By calculating indices describing the landscape, we have shown that rather than the type of habitat surrounding them, it is the arrangement of habitats that affected the abundance and richness of insects. As these results, confirmed the importance of maintaining complex landscapes to sustain both flower-visiting insect populations and crop yield, they will be of interest to fennel producers.

**Abstract:**

Agricultural landscapes are increasingly characterized by intensification and habitat losses. Landscape composition and configuration are known to mediate insect abundance and richness. In the context of global insect decline, and despite 75% of crops being dependent on insects, there is still a gap of knowledge about the link between pollinators and aromatic crops. Fennel (*Foeniculum vulgare*) is an aromatic plant cultivated in the South of France for its essential oil, which is of great economic interest. Using pan-traps, we investigated the influence of the surrounding habitats at landscape scale (semi-natural habitat proportion and vicinity, landscape configuration) and local scale agricultural practices (insecticides and patch size) on fennel-flower-visitor abundance and richness, and their subsequent impact on fennel essential oil yield. We found that fennel may to be a generalist plant species. We did not find any effect of intense local management practices on insect abundance and richness. Landscape configuration and proximity to semi-natural habitat were the main drivers of flying insect family richness. This richness positively influenced fennel essential oil yield. Maintaining a complex configuration of patches at the landscape scale is important to sustain insect diversity and crop yield.

## 1. Introduction

In a globally strained context, where food production constantly challenges biodiversity, the importance of insect activity to crop pollination is at stake, as insects are essential to the production of more than 75% of fruits, seeds, and vegetables [1,2,3]. The delivery of pollination service to crops is a known factor to improve the production of many cultivated species, such as in tomatoes [1,4] or field beans [5]. It can also increase fruit quality, for example, of apples, which are sweeter with an increased visitation rate [6], and in oilseed rape, which presents heavier seeds [7]. In economic terms, the global value of the pollination service has been estimated between USD 235 and 577 billion per year [8,9]. While the link between many crops and their insect pollinators is quite well-known, especially for some of the main mass-flowering crops such as sunflower or rapeseed [10,11], the role of insect pollinators in the agricultural production of some widespread cultivated plant species remains unclear. This is particularly the case for aromatic plants exploited for their secondary metabolites, such as medicinal hemp, lavender, mint, or fennel. Scarce studies have pointed out that self-pollination compared to cross- or open-pollination could impact the essential oil yield and secondary phytometabolite content, with major discrepancies such as self-pollination increasing essential oil yield in mint [12], while decreasing it in fennel [13]. Here, we aim to improve knowledge of the link between flower-visitor abundance and richness, and the production of essential oil among fennel (*Foeniculum vulgare* L., Apiaceae), by considering the landscape composition and configuration on the one hand, and the local management practices on fennel crops in the other hand.

In the South of France, fennel is an insect-pollinated aromatic plant of great economic interest. It is largely cultivated for its seeds, which are the richest part of the plant regarding the load in volatile aromatic compounds. Among them, *trans*-anethole, a monoterpene with an anise flavor, is the main constituent of the essential oil extracted from fennel fruits [13]. To our knowledge, few studies have described the flower-visitor community of fennel, and the results arising from the literature are widely heterogeneous. For example, as most papers present the managed honeybee *Apis* as the main fennel pollinator [14,15,16,17,18,19], a small part of the literature showed a striking importance of wild pollinators in fennel reproduction [20,21]. Among those, few authors explored the importance of these pollinators for the yield of fennel crops [14,16,17,20], and most of these studies mainly relied on the seed set as the yield measurement. Salami’s study [13] is the only one which attempted to link *trans*-anethole production and pollination, yet it considered only self- vs. cross- pollination with no regard for phytometabolite variations according to insects’ abundance and richness, or the structure of the surrounding habitats.

The diversity and patterns of land cover and local management practices are known to be the main drivers structuring insect communities in crop systems [22,23]. At the landscape scale, it is widely accepted that the diversity of habitats and the landscape complexity have a positive effect on insect communities [22,24,25]. Indeed, complex shapes and the arrangements of patches of different cover types are increasing the length of boundaries between potentially complementary resources [26], enhancing landscape heterogeneity and pollinator biodiversity [27]. In the same way, in agricultural landscapes, abundance and proximity to semi-natural habitats, useful for nesting and foraging alternative resources, can promote insect diversity [1,22,28]. As semi-natural habitats can also be a source of flower-visitors for crops [29], various authors had pointed out that increasing semi-natural habitat-crops distance implies isolation, leads to a smaller flower-visitation rate [30,31], and decreases pollination service [32,33]. At a more local scale, the size of parcels [34,35], and the use of agrochemicals [36] are widely known to be strong markers of agricultural intensification that negatively affect flower-visiting insects [37,38]. All components of agricultural intensification, expressed at a landscape or a local scale, are related and partially additive [22], and must be considered together to understand the link between agricultural intensification, insect community structure, and crop yields.

To fill in the knowledge gap linking flower-visitor assemblages, pollination, and yield of fennel, we investigated flying insect community foraging at the edge of fennel crops using pan-trap. Using structural equation models, we explored how fennel yield essential oil can be linked to (1) the local management practices (insecticide, herbicide and fungicide use; irrigation; fertilizer and the size of parcels), (2) the landscape composition (land cover; distance to semi-natural habitats) and (3) the landscape configuration (interspersion and juxtaposition index) through the impact of these variables on the abundance and family richness of flying insects. Our results are discussed with a particular emphasis on the importance of maintaining complex landscapes to sustain both abundant insect populations and crop yield. 

## 2. Materials and Methods

### 2.1. Study Area and Plant Species

The study was carried out in the Mediterranean Basin, in the area named “*Plateau de Valensole*” (Alpes-de-Haute-Provence, South of France), on fennel crops cultivated for a local anise spirit production, called “*pastis*”. The cultivated aromatic variety of *Foeniculum vulgare* is developed by the company Pernod-Ricard^®^ and called “*Jupiter*”. As in many Apiaceae species, a protandry is supposed in fennel flowers (centripetal development, i.e., earlier anthers occur in outer flowers of umbels and outer umbellets within an umbel). Each flower contains five stamens, with a nectar-bearing surface at their base. Each fertilized fennel flower may lead to a fruit, which is a di-achene, and each achene can hold one seed. According to the sowing date, fennel can bloom from mid-June to October.

### 2.2. Experimental Field Design

We selected ten fennel fields representative of the diversity of landscapes of the “*Plateau de Valensole*”. For each field, we selected one edge, which will represent our experimental sites, for a total of 10 sites. Five sites were immediately adjacent to woody semi-natural habitat (SNH) and five were not. The following were considered as SNH: hedgerows, woody boscages, and abandoned patches of truffle oaks. In the South of France, the fennel is sown at two periods of the year, resulting in two main flowering periods. The first flowering period begins at the end of June, peaks during July, and is harvested in early August. The second begins at the end of July, maximum blooming is in August and harvest occurs in mid-September. All the fields are, therefore, blooming synchronously between the last fortnight of July and the first fortnight of August. Among our 10 fields, five were issued from the first sowing period (three in SNH vicinity edge and two not) and five from the second one (two in SNH vicinity edge and three not). 

### 2.3. Insect Sampling

**Figure A1:**
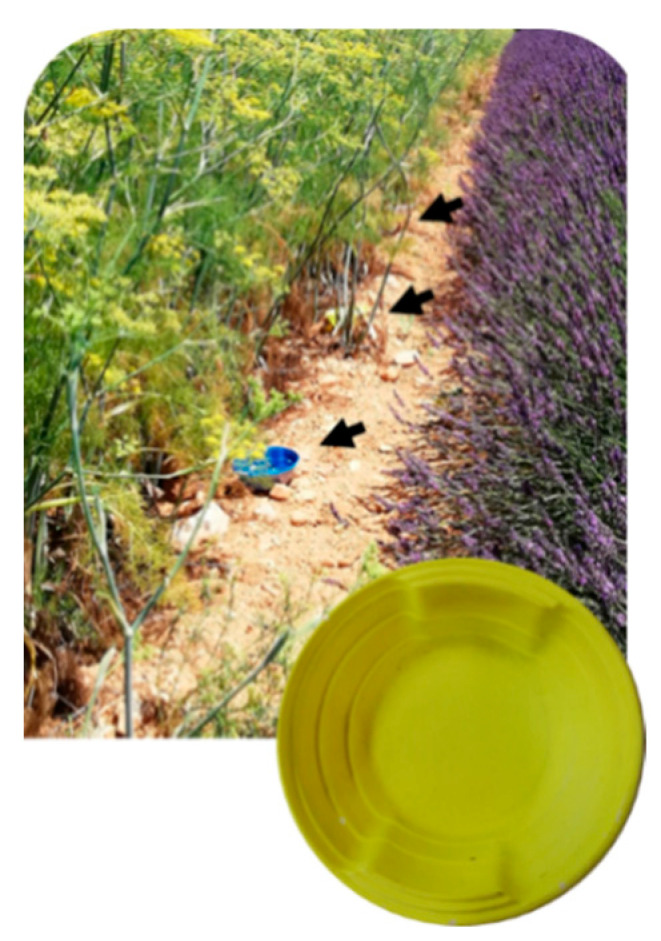
Pan-trap type used to insect captures.

Insects were caught through pan-trapping [39] (Appendix A, Figure A1). Pan-traps are very efficient at capturing insects without observer bias and are a good tool to understand the impact of landscape and local variables on insect communities, but are not a good method to precisely identify the pollinators of a given crops [39]. Three colored pan-traps (one blue, one white and one yellow) were placed, aligned and separated from each other by two meters, in each experimental site. The pan-traps were filled with soapy water and left 24 h on site. Each site was sampled three times between June and September, during the respective flowering period of each field. In total, we collected 90 pan-trap samples. All insects were collected and stored in 70% ethanol. Then, they were identified to the lowest possible taxonomic level and at least to the family level. For this study, we only kept flower-visiting insects, while parasite or predator insects were left out. Damaged insects were classified as “non-identified insects”.

### 2.4. Landscape Composition and Configuration, and Local Management Practices

To analyze the landscape surrounding of each site, we modelled 1 km radius buffers using online GPR data (graphic parcel register), photo-interpretations on orthophotos, and field assessments (QGIS software 3.2 madeira). This radius was chosen because it encompasses most of the foraging distance of flying insects we trapped [40,41,42,43]. For each buffer, we categorized nine different land cover types, namely: ‘urban areas’, ‘water surface’, ‘SNH’, ‘MFC (mass flowering crops)’, ‘meadow’, ‘fallow’, ‘non-flowering crops’, ‘orchards’ and ‘other habitats’. Buffers were rasterized with a spatial resolution of 5 m × 5 m. We then calculated the proportion of each land cover (in percentage) at the 1 km radius. Then, using the Fragstats software 4.2.1 [44], we calculated the interspersion and juxtaposition index (IJI) for each buffer. IJI is a measure of the landscape spatial configuration of habitats patches. This index tends toward 0 when patches of different land cover types are not adjacent to each other and are unequally distributed within a landscape. On the contrary, IJI tends toward 100 when patches of different land cover types are adjacent to each other’s and share borders [45]. In other words, IJI measures habitat aggregation and land cover types mixing through the contiguity of the patch edges of different land cover types [25]. Finally, as isolation from semi-natural habitats is known to influence the abundance and richness of flower visitors [30,31], we measured the distance from each of our sampling site to the closest SNH (in meters).

Regarding the local management practices, from each farmer, we obtained information about irrigation (L/ha), fertilizer use (Kg/ha), weed, insect and fungus controls input (L/ha) (Appendix B, Table A1). Finally, as the increasing size of habitat patches has a negative effect on insect abundance [34,35], each fennel field area (ha) was measured (Appendix B, Table A1).

The environmental variable selection for modelling was assessed by generating a principal component analysis, a correlation matrix and correlation tests, excluding the collinear metric. This selection led us to keep, among all explanative variables, the percentage of SNH and the interspersion and juxtaposition index (IJI) at the landscape scale (at 1 km radius), the distance from our sites to the closest SNH (m), the parcel size (ha) and the total insecticide quantity (L/ha).

### 2.5. Essential Oil Yield

The amount of essential oil (EO) obtained from each field was provided by farmers. Fennel EO comes from the hydro-distillation of aerial parts of the plant, which contain the “*trans*-anethole” as a major component (*Jupiter* variety of fennel contains at least 70% of *trans*-anethole in its essential oil). During harvest, cut plant material was immediately collected and stored in closed tanks that were then used for hydro-distillation in cooperative stills (maximum three hours after harvest). Steam-distillation of fresh plant material lasted for two hours. Because the quantity of essential oil obtained is dependent on the parcel size, we expressed the yield in kilogram of essential oil per hectare (EO kg/ha).

### 2.6. Data Analysis 

We tested the causal structure of selected variables using a path-analysis (structural equation modelling (SEM) [46]). SEM provides a way to model indirect effects and allowed us to obtain causal relationships, and not just correlations, between variables [46] joining multiple predictor and response variables in a single network. “Piecewise SEM” package (R version 4.0.2 2020-06-22 [47]) provides a method of assessing the goodness-of-fit based on Shipley’s test of directed separation that combines the *p*-values of all independence claims in Fisher’s C [46,48,49,50]

$$C=-2\sum_{i=1}^{k}ln(pi)$$

where *pi* is the *i*^th^ independence claim in a basis set consisting of *k* claims. The *C* statistics can be then compared to a *χ*^2^ distribution with 2*k* degrees of freedom [46,48,49]. The hypothesized relationships are consistent with the data when the collection of the relationships represented by *C* could have occurred by chance, in which case the *p*-value for the *χ*^2^ test is greater than the threshold 0.05, and the path model is rejected if the *p*-value is <0.05 [46,48,49].

For our statistical analyses, we pooled the three capture sessions on each site. We thus obtained three samples (from each pan-trap color) for each one of the ten sites. A statistical sample is thus the ‘parcel/pan-trap’ pair (N = 30). 

We used generalized linear mixed effect models (glmer) with a Poisson error distribution including a random effect (experimental site identity) to explain the (1) abundance and (2) family richness of flying insects per sample. Predictor variables were the percentage of SNH and the IJI index at the landscape scale, the distance from each sample sites to closest SNH, the parcel size and the insecticide amount. 

Regarding the essential oil yield, we also used linear mixed-effect models, but with a Gaussian family including a random effect on pan-trap color (glmer). Predictor variables were the abundance and family richness of flying insects. The best models were selected using the ‘dredge’ function (MuMin package of R version 4.0.2 2020-06-22) based on a comparison of their corrected Akaike Information Criterion-AICc, and subsequently included in the path-analysis. We always selected the model with the lowest AIC to set the path-analysis.

We performed the path-analysis using the “Piecewise SEM” package version 2.1.0 (R version 4.0.2 2020-06-22) [46] and following the Shipley Method [48,49]. Missing paths were added before interpretations of the final path-analysis coefficients. The residuals of all models were checked for homoscedasticity and normality and we checked for collinearity using variance inflation factors (VIFs) and the ‘car’ package. 

## 3. Results

We captured 2036 flying insects, representing 38 families (Appendix C, Table A2): 839 Hymenoptera belonging to 17 families, including five families and 662 individuals of bees, 613 Coleoptera belonging to 10 families, 462 Diptera from nine families, and 14 Lepidoptera from three families. The most abundant family was the Apidae, forming 21% of captures, mostly represented by the managed honeybee *Apis mellifera* L. (97% of Apidae), followed by two families of Coleoptera, the Meloidae (12%) and the Mordellidae (10%).

Regarding the land cover, most of the site’s surroundings were SNH (42.6% ± 20.6) followed by mass flowering crops (*Lavandula hybrida, Salvia sclarea, Helichrysum italicum*; 24.6% ± 18.8) and then by other crops (12.9% ± 8.7). The other land cover types represented less than 5% of the remaining area within our buffers.

### 3.1. Impact of Landscape Composition and Configuration and Local Management Practices on Flower-Visiting Insects

**Figure 1:**
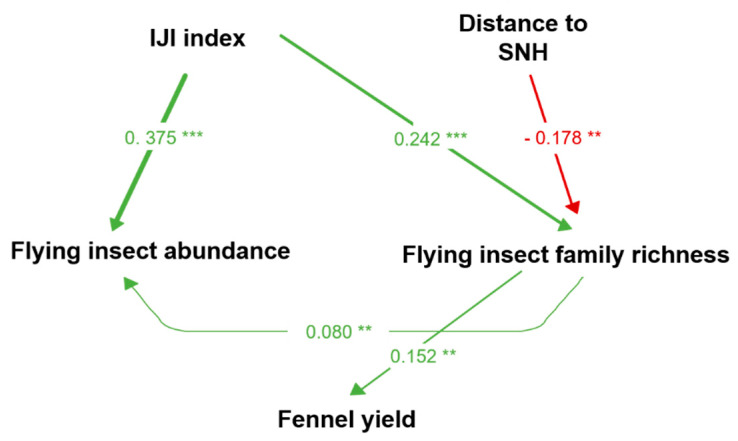
Path diagram of the structural equation modelling showing the drivers of fennel insect communities and yield (Fisher’s *C* = 10.575, *k* = 8, *p* > 0.05). The width of arrows depends on effect’s size. *** *p* < 0.001; ** *p* < 0.01. (Arrow color helps visualize the sign of the effect, green: positive effect; red: negative effect). See Appendix D for details of the path-analysis settings.

After the selection of best model and the addition of missing path(s) (Appendix C), the path-analysis explained our data adequately (Fisher’s *C* = 10.575, *k* = 8, *p* = 0.227; Figure 1, Table 1).

**Figure 2:**
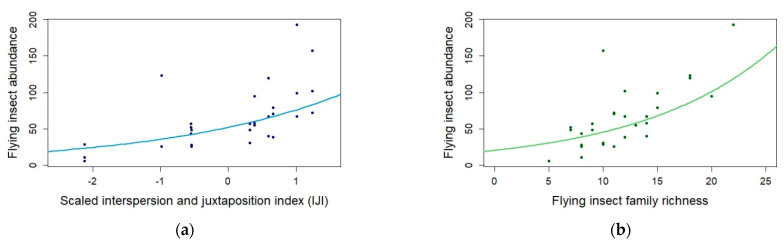
(**a**) Flying insect abundance variations according to the interspersion and juxtaposition index IJI (blue; E = 0.3747, *p*-value < 0.001); (**b**) Flying insect family richness (green; E = 0.0798, *p*-value < 0.01) - Glmer with Poisson family; N = 30.

The path-analysis showed that flying insect abundance was positively influenced by landscape configuration (higher IJI index; E = 0.3747, *p*-value < 0.001 ***; Table 1, Figure 1 and Figure 2a), and flying insect family richness (E = 0.0798, *p*-value <0.01 **; Table 1, Figure 1 and Figure 2b).

**Figure 3:**
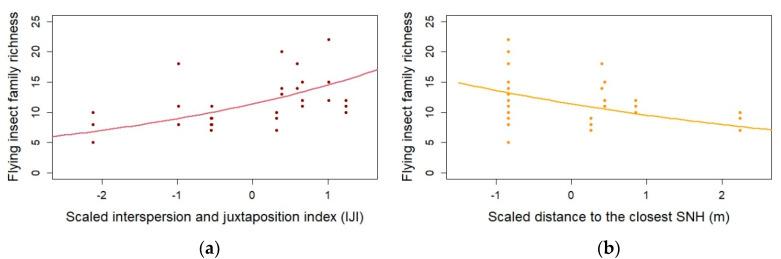
(**a**) Flying insect family richness variations according to the interspersion and juxtaposition index IJI (red; E = 0.2419, *p*-value < 0.001), and (**b**) distance to semi-natural habitat SNH (yellow; E = 0.0798, *p*-value < 0.01)—Glmer with Poisson family; N = 30.

Flying insect family richness was positively influenced by landscape configuration (higher IJI index; E = 0.2419, *p*-value < 0.001 ***; Table 1, Figure 1 and Figure 3a), and negatively affected by an increase in the distance to semi-natural habitat (SNH; E = −0.1777, *p*-value < 0.01 **; Table 1, Figure 1 and Figure 3b).

### 3.2. Insect Impact on Fennel Yield

**Figure 4:**
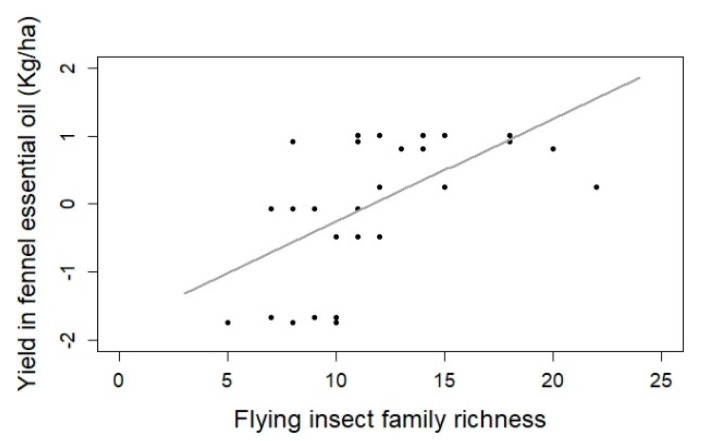
Essential oil yield increases with flying insect family richness-E = 0.1515, *p*-value < 0.01 (glmer with Gaussian family; N = 30).

The path-analysis showed that the production of fennel essential oil was positively related to an increase in the family richness of flying insects (E = 0.1515, *p*-value < 0.01 **; Table 1, Figure 1 and Figure 4).

## 4. Discussion

*Foeniculum vulgare* in our system seems to be a generalist plant species, attracting a great diversity of insects along its edges. The main insect trapped belonged to the Apidae bees, almost entirely represented by the managed honeybee *Apis mellifera*, followed by Coleopterans. Increasing distance to the closest SNH decreases insect family richness. We showed that landscape configuration mediates insect communities more than SNH proportion. Surprisingly, we found no effect of the local agricultural practices on insect communities. We confirmed that the family richness of flying insects plays a major role in fennel crops, positively driving the fennel essential oil yield.

### 4.1. Flower Visitor Community of Fennel Crops

We found a wide richness of insect families at fennel edges with 38 different families trapped, showing that *Foeniculum vulgare* is susceptible to attracting a large diversity of insects. An overview of the few papers published about the flower-visitors of fennel illustrates that it can indeed attract bee, wasp, syrphid fly, moth, butterfly and coleopteran species [13,14,15,16,17,18,19,20,21]. In our pan traps, the western honeybee (*Apis mellifera*) was the most abundant insect, representing 21% of the total abundance. This is consistent with intense beekeeping activity on the “*Plateau de Valensole*” due to the abundance of lavender (*Lavandula hybrida*) crops in the area. To produce an economically profitable lavender honey, many beekeepers install their hives during the lavender’s flowering period, which overlaps with the beginning of the fennel flowering. *Lavandula hybrida* is known to produce nectar but no pollen because it is a sterile hybrid. Honeybees looking for pollen grains (for the development of their brood) can find this resource on fennel flowers. This high number of honeybees could be under-evaluated, as pan trapping is known to poorly capture honeybees. Previous studies have shown that several *Apis* species (*A. florea, A. cerana, A. dorsata, A. mellifera*) could visit and pollinate the fennel [14,15,16,17,19], and we hypothesize here that the presence of *Apis mellifera* could be profitable for the fennel yield. It could be interesting to explore the complementarity between fennel and lavender crops in future studies, both for the feeding requirement of honeybees and the pollination of fennel. Many wild bees were also found in traps, especially Halictidae and Andrenidae, showing that fennel might be attractive for wild bees too. Previous studies have shown that fennel is visited by a wide diversity of wild bees [20,21] and future studies could investigate their relative efficiency for the pollination of fennel and its yield. 

The other most abundant families were Coleopterans: Meloidae (12%) and Mordellidae (10%). The role of Coleopterans in the pollination of fennel remains unclear because, when visiting flowers, they are mainly grazing pollen [16,21]. 

Despite their reported importance as flower visitors of fennel in the literature [14,15,16,18,20,21], only a few wasps (64 specimens, representing 2.8%), and syrphids (34 specimens, representing 1.5%) were trapped. However, this discrepancy could be explained by differences in capture methods and environmental conditions.

### 4.2. Impact of Landscape Composition and Configuration on Fennel Flower-Visiting Insects

Landscape composition [27,30] and configuration [28] are important drivers of insect assemblages. Semi-natural habitats are known to offer shelters for insects, including a wide range of nesting sites and a diversity of floral resources [51,52,53]. Therefore, the literature widely reports that increasing SNH proportion in the landscape has a positive effect on insect abundance and/or richness [27,37,51,54,55]. Our results did not link SNH proportion and insect abundance/richness. This has already been shown previously, with SNH having no effect [35,56] or a negative effect [25] on bee abundance and diversity. an explanation could lie in the type of SNH considered (with herbaceous being more attractive than woody) [57], or because of a dilution effect due to other mass-flowering crops attracting insects [35,58]. However, even if we found no effect of the proportion of SNH on insects, we have shown that the increasing distance to SNH is negatively impacting flying insect richness in fennel fields. Being in the vicinity of SNH has been previously acknowledged to increase abundance [51,59,60], richness [30,37,51], visitation rate [30,33], functional diversity [31], turnover of flower-visitors [29], fruit production [33], and crop quality [55]. 

The interspersion and juxtaposition index relies to the configuration of landscape habitats, i.e., the arrangement of the different habitats’ patches in the landscape [25,35]. This index thus reports the patch mixing and length of adjacencies between habitat type. In this way, an increase in IJI conveys an increase in the linear edges’ length between crops. Some studies showed that patch-mixing positively influences bee assemblages [25,28], others found a negative effect of IJI on insect community [61,62]. We have shown that increasing IJI has positive impact on flying insect abundance and richness. Indeed, an increase in the linear edges length between crops may facilitate access to varied resources for insects and can be complementary for their life cycles [35,63]. For example, many local wild bee species are nesting in pre-existing cavities in dead wood [64], and such species could benefit from being close to both lavender (for nectar) and fennel (for nectar and pollen) while foraging. In this way, a high IJI is beneficial for these species.

### 4.3. Impact of Local Management Practices on Flower-Visiting Insects

Beside landscape composition and configuration, agricultural practices can impact insect abundance and richness; effects at the local and landscape scale are often additive [22,28]. An increase in croplands in a landscape is known to negatively influence insect abundance and diversity [37,65], particularly when paired with the use of insecticides [36] and big crop size [34,35]. Surprisingly, we did not show any link between local management practices and markers of agricultural intensification on fennel insect abundance and family richness. We propose four hypotheses to interpret this result. First, the negative effect could depend on the type of agricultural intensification, as in Le Féon et al. [37], who found different responses of bee assemblage between fallow and grazing lands. Secondly, agricultural intensification can be mitigated by other local or landscape features, such as surrounding habitat quality, according to Kennedy et al. [28]. Thirdly, insect abundance and richness variations could be hidden because a critical threshold value in intensification was reached years ago; therefore, the current insect community would represent a remnant of the historical biodiversity, composed of tolerant species. Finally, the use of insecticide might be below the threshold that affects insect communities, with regard to amount or spreading periods [66]. We cannot settle this point at present, but further studies are in progress (see also Appendix D for further discussions relative to this point). 

### 4.4. Insect Impact on Fennel Yield 

This study is the first one linking fennel essential oil yield variations to insect abundance and family richness. We showed here that family richness of insects trapped along fennel crops was positively linked with essential oil yield. If we keep in mind that, in fennel, the fruits are the part of the plant containing the highest amount of essential oil, this result is in line with the substantial literature showing the importance of a high diversity of flower-visiting-insect for the yield, quantity and quality of seeds and fruits, and their market value [28,34,67,68,69]. This has notably been emphasized in other mass-flowering crops (e.g., oilseed rape [11], sunflower [10]) or orchards (e.g., apples or/and pears [6,70]). From ecological studies, we know that a community with diverse and complementary traits can enhance the ecosystem functions [68,71,72]. This is notably the case, for example, for pumpkin [73] or apple orchards [74]. Here, increasing flower-visitor richness involves increased diversity of insect traits (different mouthparts, foraging behavior variation, hour and date of insect activity) and matches the complementary hypothesis of functional diversity [68,71,72]. 

Here, we could not link essential oil yield to insect abundance. This makes sense because a great abundance is not necessarily a guarantee of pollination; a flying insect found in the crop and surroundings, or even flower visitor, is not always an effective pollinator [75,76]. Indeed, further studies could be completed by net captures on fennel flowers, integrating a direct measure of pollination (i.e., the quantity and quality of pollen deposited on fennel flowers and pollen tubes’ growth), and exploring insect morphological and behavioral traits.

## 5. Conclusions

Our study is the first to quantify fennel essential oil variations according to the family richness and abundance of insects trapped along fennel crops. We highlighted that the landscape configuration and the vicinity to semi-natural habitats drives insect family richness. Further studies should investigate the ecological, morphological and behavioral traits of fennel-flower-visitors for a better understanding of the link between insects and fennel yield. It would also be interesting to go further in the measurement of the fennel yield, exploring various phytometabolite variations (especially the most valuable ones). As these results confirmed the importance of maintaining complex landscapes to sustain both flying insect populations and crop yield, they will be of interest to fennel managers and producers. 

## Figures and Tables

**Table 1:** Path coefficients of the structural equation modelling showing the drivers of fennel insect communities and yield (Fisher’s *C* = 10.575, *k* = 8, *p* > 0.05). *** *p* < 0.001; ** *p* < 0.01.

Response	Predictor	Estimate	Std. Error	DF	Crit. Value	*p*. Value
Insect abundance	IJI	0.375	0.089	30.000	4.193	<0.0001 ***
Insect abundance	Insect family richness	0.080	0.024	30.000	3.278	0.001 **
Insect family richness	IJI	0.242	0.062	30.000	3.902	<0.0001 ***
Insect family richness	Distance to SNH	−0.178	0.062	30.000	−2.853	0.004 **
Fennel yield	Insect family richness	0.152	0.039	26.556	12.024	0.002 **

## Data Availability

We make the data available on request; please contact the corresponding author L.S. (lucie.schurr@gmail.com).

## Appendix B

**Table A1:** Intensification parameter raw data.

Parcel ID	Irrigation (L/ha)	Insecticides (L/ha)	Herbicides (L/ha)	Fungicides (L/ha)	Fertilizer (Kg/ha)	Parcel Size (ha)
“FNO2”	3,000,000	0	3.500	1.500	300	9.200
“FNO3”	0	0.200	2.000	0.500	550	2.480
“FNO5”	0	0	3.500	0.000	40	2.820
“FNO6”	3,200,000	0.125	2.800	1.000	400	5.890
“FNO8”	1,050,000	0.100	2.625	1.000	500	9.220
“FNO9”	2,100,000	0	2.800	1.500	280	4.860
“FNO15”	600,000	0	1.500	0.500	240	1.390
“FNO16”	600,000	0	1.500	0.500	240	2.540
“FNO17”	0	0	2.800	0	100	1.100
“FNO18”	0	0.125	2.800	1.500	450	1.760

## Appendix C

**Table A2:** Insect families caught.

Insect Family	N
**Hymenoptera**	**839**
Apidae	493
Halictidae	111
Vespidae	64
Sphecidae	54
Andrenidae	38
Pompilidae	17
Colletidae	12
Sapigydae	10
Megachilidae	8
Ichneumonidae	7
Scoliidae	7
Undetermined	6
Formicidae	5
Chysididae	3
Tenthredinidae	2
Drynidae	1
Evaniidae	1
**Coleoptera**	**613**
Meloidae	281
Mordellidae	225
Buprestidae	36
Cetoniidae	19
Melyridae	19
Cerambycidae	12
Oedemeridae	12
Cleridae	5
Coccinellidae	3
Chrysomelidae	1
**Diptera**	**462**
Sarcophagidae	190
Anthomyiidae	93
Bombyliidae	64
Tachinidae	54
Syrphidae	34
Conopidae	17
Stratyomyidae	7
Therevidae	2
Bibionidae	1
**Lepidoptera**	**14**
Undetermined	8
Nymphalidae	3
Zygaenidae	3

## Appendix D. Path-Analysis Setting Details and Discussion

In the literature, as a rule of thumb, if the delta between two values of AIC values is greater than 2, the model with the lower AIC shows a significant improvement in parsimony [77]. Here, for our path analyses, when several models were discriminated with a delta AICc < 2, we systematically chose the models with the lowest AICc.

### Appendix D.1. Impact of Landscape Composition and Configuration on Flower-Visiting Insects

Preliminary model selection led to three equivalent models to explain the abundance of flying insects (Table A3). According to these models, the insect abundance is positively influenced by the landscape configuration (higher IJI index), and the percentage of SNH (Table A3). The abundance is, however, negatively influenced by the increased distance to SNH.

We selected the model with the lowest AIC to set the path-analysis, i.e., the model which explained abundance according to the IJI index. We added the missing path explaining the insect abundance by the insect family richness to the selected model.

**Table A3:** Insect abundance model selection based on the comparison of their corrected Akaike Information Criterion-AICc with the dredge function (MuMin package of R version 4.0.2 2020-06-22).

(Int)	SNH Dist.	IJI	% SNH	Pesticide	Parcel Size	df	logLik	AICc	Δ	Weight
3.953	-	0.532	-	-	-	4	−138.459	286.500	0.000	0.254
3.954	-	0.636	0.166	-	-	5	−137.312	287.100	0.610	0.181
3.954	−0.122	0.581	-	-	-	5	−137.621	287.700	1.220	0.133
3.953	-	0.515	-	0.082	-	5	−138.019	288.500	2.020	0.089
…	…	…	…	…	…	…	…	…	…	…
3.955	-	-	-	-	-	3	−147.459	301.800	15.320	0.000

Preliminary model selection also led to three equivalent models to explain the family richness of flying insects (Table A4). According to these models, the family richness was positively influenced by an increase in the IJI index and the percentage of SNH (Table A4). The family richness was, however, negatively affected by the distance to the closest woody SNH (Table A4). We selected the model with the lowest AIC to set the path-analysis, i.e., the model which explained richness according to the IJI index, and the distance from each sample site to the closest SNH. There was no missing path to add to this selected model.

**Table A4:** Flying insect family richness model selection based on the comparison of their corrected Akaike Information Criterion-AICc with the dredge function (MuMin package of R version 4.0.2 2020-06-22).

(Int)	SNH Dist.	IJI	% SNH	Pesticide	Parcel Size	df	logLik	AICc	Δ	Weight
2.434	−0.178	0.242	-		-	4	−74.169	157.900	0.000	0.265
2.430	−0.155	0.220	-	0.077	-	5	−73.165	158.800	0.890	0.170
2.431	−0.194	0.197	-	-	0.074	5	−73.664	159.800	1.890	0.103
2.433	−0.165	0.265	0.046	-	-	5	−76.959	160.400	2.480	0.077
…	…	…	…	…	…	…	…	…	…	…
2.438	-	-	-	-	-	2	−80.264	165.0	7.03	0.008

In models 2 and 3 (higher AICc but without a delta AICc > 2), parcel size and pesticide use seemed to have no effect on insect abundance and a positive effect on insect family richness. Sihag ([66]) already showed the positive synergistic effect of insect pollination and use of pesticides on seed yield. It has been shown that different pesticides could have different effects on different organisms [78]. The increase in parcel size could also have a positive effect on insect abundance [79] and richness as it increases the floral display of mass-flowering crops and, thus, floral resource availability [80], leading to an increase in foraging efficiency [81] and decrease in foraging energetic cost [82]. As our studied crops were managed with sustainable managing practices (sustainable agriculture), we cannot exclude that the amount of pesticide used might be below a threshold that affects insect communities.

### Appendix D.2. Insect Impact on Fennel Yield

Only one model was selected to explain the fennel essential oil yield quantity (Table A5). According to this selected model, richness but not abundance positively influences fennel essential oil yield (Table A5).

**Table A5:** Fennel essential oil yield model selection based on the comparison of their corrected Akaike Information Criterion-AICc with the dredge function (MuMin package of R version 4.0.2 2020-06-22).

	(Int)	Abundance	Family Richness	df	logLik	AICc	Δ	Weight
3	−1.772 × 10^00^	-	0.152	4	−39.286	88.200	0.000	0.897
1	−5.551 × 10^−17^	-	-	3	−42.850	92.600	4.450	0.097
4	−1.909 × 10^00^	−0.005772	0.194	5	−43.006	98.500	10.340	0.005
2	−4.402 × 10^−1^	0.006850	-	4	−46.115	101.800	13.660	0.001
